# Investigating the Dual Role of Trace and Toxic Elements in Pregnancy-Related Health Outcomes

**DOI:** 10.3390/ijerph22091423

**Published:** 2025-09-12

**Authors:** Thaveesak Sai-ong, Donrawee Waeyeng, Tanaporn Khamphaya, Yanisa Rattanapan, Warinya Hnoocham, Katesiri Samaphong, Soisungwan Satarug, Supabhorn Yimthiang

**Affiliations:** 1 Environmental, Safety Technology and Health Program, School of Public Health, Walailak University, Nakhon Si Thammarat 80160, Thailand; thaveesaksaiong@gmail.com (T.S.-o.); donrawee.wae@gmail.com (D.W.); tanaporn.kh@wu.ac.th (T.K.); 2 Department of Environmental Health, School of Public Health, Walailak University, Nakhon Si Thammarat 80160, Thailand; 3 Excellence Center for Public Health Research, Walailak University, Nakhon Si Thammarat 80160, Thailand; 4 Department of Occupational Health and Safety, School of Public Health, Walailak University, Nakhon Si Thammarat 80160, Thailand; 5 School of Allied Health Sciences, Walailak University, Nakhon Si Thammarat 80160, Thailand; 6 Hematology and Transfusion Science Research Center, Walailak University, Nakhon Si Thammarat 80160, Thailand; 7 The Center for Scientific and Technological Equipment, Walailak University, Nakhon Si Thammarat 80160, Thailand; 8 Faculty of Science and Industrial Technology, Prince of Songkla University, Surat Thani 84000, Thailand; 9 Centre for Kidney Disease Research, Translational Research Institute Woolloongabba, Brisbane, QLD 4102, Australia

**Keywords:** trace elements, toxic elements, ICP-OES, pregnancy outcomes

## Abstract

Maternal health during pregnancy can be influenced by exposure to essential trace and toxic elements, notably cadmium, lead, chromium, and arsenic. Using inductively coupled plasma optical emission spectrometry, this study determined blood concentrations of toxic elements together with zinc, copper, and iron, which are nutritionally essential, in 200 pregnant women who attended the antenatal care at Thasala Hospital, Nakhon Si Thammarat, between January and July 2023. Associations of maternal clinical outcomes with the measured blood elemental composition were evaluated by Spearman’s rank correlation analysis. Iron showed the highest concentration among trace elements (75,178 ± 12,045 µg/dL), followed by zinc (1189.20 ± 211.38 µg/dL) and copper (294.72 ± 67.19 µg/dL). Among the toxic elements, lead had the highest concentration (5.59 ± 1.61 µg/dL), followed by chromium (2.80 ± 1.47 µg/dL), with arsenic and cadmium having the lowest concentrations. Synergistic associations were observed among lead, zinc, and iron. Blood lead concentration correlated inversely with hematocrit, while blood arsenic and blood cadmium both showed inverse association with urine glucose. Plasma glucose concentration varied directly with zinc and iron. These findings indicate effects and interactions of essential versus toxic elements on the health of pregnant women. They underscore the need to continue research into the strategies to minimize the impact of toxic elements and to further improve the nutritional status of zinc and iron during pregnancy.

## 1. Introduction

Exposure to trace and toxic elements, especially during pregnancy, remains to be an issue for public health concern [1]. Pregnant women are particularly vulnerable to insufficient intake of trace elements and exposure to toxic elements, both of which can have adverse effects on maternal health and fetal development [2]. During pregnancy, the physiological demand for trace elements iron (Fe), zinc (Zn), and copper (Cu) are increased markedly. Notably, Zn, Cu, and magnesium (Mg) deficiencies have been shown to impact prenatal outcomes [3]. They have been associated with infertility, pregnancy loss, congenital abnormalities, pregnancy-induced hypertension, placental abruption, preterm rupture of membranes, stillbirths, and low birth weight [4,5,6].

Anemia is one of the most prevalent disorders in pregnant women; in 2023, it affected approximately 35% of women, with 30–60% having Fe deficiency [7]. Global trends include Zn deficiency in 8% of rural Pakistani women [8], 53% of women in Ethiopia’s Sidama region [9], and 9% of women in Ireland [10]. In the northern region of Thailand, specifically in Chiang Mai, more than 50% of pregnant women experienced a Zn deficit during late gestation despite receiving supplementation [11]. Among women in Pattani Province, the southern region, the prevalence of Fe deficiency and anemia was 34.4% and 37.8%, respectively [12]. The 2020 Thai Dietary Reference Intake recommends a daily Zn intake of 10.8 mg, in conjunction with universal Fe supplementation; however, existing standards and prenatal supplements may still be inadequate [13]. Furthermore, insufficient intake levels of essential trace elements might be exacerbated by exposure to toxic elements.

Excessive exposure to arsenic (As), lead (Pb), cadmium (Cd), and mercury (Hg) together with inadequate intake of essential trace elements, Zn, Cu, and Fe can produce unfavorable pregnancy outcomes [14,15]. Current evidence suggests synergistic and/or antagonistic effects of these elements. For instance, the intestinal absorption of Pb and its toxicity have been shown to be diminished by Zn and Fe [16]. Conversely, the toxicity of Cd and As was enhanced by Zn and Fe deficiencies [16]. Concerningly, Pb is known for its teratogenicity because it can readily cross the placenta and reach the fetus [17].

In a study from Nigeria, maternal blood Pb levels were linked to reduced birth weight and increased risk of preterm delivery [18]. In a Chinese study, maternal exposure to Pb was associated with an elevated risk of premature rupture of membranes [19]. In the study from Mexico, maternal exposure to Pb was linked to gestational hypertension and adverse neonatal outcomes [20]. Thai research data showed that the prevalence of pregnant women with blood Pb levels exceeding a permissible level (>5 mg/dL) was 42.5% [17]. Thus, excessive maternal exposure to Pb remains a public health issue in Thailand. Data on maternal exposure to toxic elements other than Pb are limited. Such pregnancy-specific exposure data are essential for the development of specific target public health measures. Accordingly, the present study aimed to investigate blood concentrations of essential trace elements (Zn, Cu, Fe) and toxic elements (Cd, Pb, Cr, As) in pregnant women and evaluate their association with pregnancy-related clinical indicators.

## 2. Materials and Methods

### 2.1. Study Design and Sample Population

This cross-sectional study was conducted at the Obstetrics and Gynecology Department of Thasala Hospital, Nakhon Si Thammarat Province, Thailand, between January and July 2023, enrolling a total of 200 pregnant women. Eligible participants were women aged 18 years or older who attended antenatal care (ANC) at Thasala Hospital, had no pre-existing chronic medical conditions such as diabetes or hypertension, and had no history of major pregnancy complications. Women were excluded if they had severe anemia requiring transfusion, known hematologic disorders, or incomplete clinical and laboratory records.

### 2.2. Data Collection Tools

Data were collected using a structured and validated questionnaire. The questionnaire covered (1) sociodemographic variables—age, gestational BMI, education, gravidity, and occupation. (2) Clinical parameters—blood pressure, hematocrit, fasting plasma glucose (FPG), oral glucose tolerance test (OGTT) at 1, 2, and 3 h, urine protein, and urine glucose.

### 2.3. Clinical and Laboratory Variables

The validated questionnaires were used to gather data on mother occupation, education level, sociodemographic factors determinants (age, gestational age, and gestational body mass index, or G-BMI), and clinical parameters, including complete blood count (CBC), oral glucose tolerance testing (OGTT), systolic blood pressure (SBP), diastolic blood pressure (DBP), obstetric history, including gravida (number of pregnancies; G), urine glucose and urine protein.

The venous maternal blood sample (3 mL) was collected at the time of visit to the ANC clinic. The blood samples were obtained by venipuncture and collected in ethylenediaminetetraacetic acid (EDTA) tubes. EDTA tubes were used as anticoagulants to prevent blood coagulation. The test tubes were placed in crushed ice and stored at −20 °C until required for analysis.

### 2.4. Determination of Trace and Toxic Elements in Maternal Blood

After being fully thawed at room temperature, the frozen blood samples were homogenized. A total of 2 mL of concentrated nitric acid and 1 mL of 30% hydrogen superoxide were used to digest 1 mL of blood. The samples’ solutions were then diluted with 15 mL of distilled water to detect elements [21,22]. Inductively coupled plasma optical emission spectroscopy (ICP-OES, Varian 730. Agilent Technologies, Santa Clara, CA 95051, U.S.) was used to determine the levels of trace elements (Zn, Cu, and Fe) and toxic elements (Cd, Pb, Cr, and As). It was operated under the proper conditions, which included selecting the appropriate wavelength for each element (Table 1) with plasma argon flow rates of 12 L/min, auxiliary argon flow rates of 1.0 L/min, and nebulizer argon flow rates of 0.6 L/min, integration time of 100 s, and read delay of 15 s. Every chemical product employed was of the caliber of an analytical reagent. Solutions in 18.2 MΩ cm deionized water were produced. The standard XVI multi-element ICP standard solution from Merck KGaA, Darmstadt, Germany, was used to create calibration standards for each element. Analysis did not include values below the limit of detection (LoD). The concentrations of elements are expressed as mg/L.

### 2.5. Statistical Analysis

Data were analyzed using SPSS version 28. Continuous variables were tested for normality using the Shapiro–Wilk test. As the data were not normally distributed, nonparametric statistical methods were applied. Spearman’s rank-order correlation was used to assess the associations between toxic element concentrations and clinical variables. The Kruskal–Wallis test was applied when there were more than two independent groups in the quantitative variable comparisons. If a statistically significant difference is detected, Dunn’s post hoc test with Bonferroni correction is applied to determine which specific group accounted for the difference. A *p*-value < 0.05 was considered statistically significant. Descriptive statistics were presented as mean ± SD or median, and categorical variables as frequencies and percentages.

## 3. Results

### 3.1. General Characteristics

A total of 200 pregnant women participated in the study, and Table 2 lists all their socio-demographic details. The ages of the pregnant women ranged from 18 to 45 years, with an average age of 30.2 ± 7.5 years and a gestational BMI of 27.5 ± 5.9 kg/m^2^, and 41.0% of them were in the third trimester. The majority had at least three pregnancies (44.5%) and had finished high school (52.0%). Housewives represented 33.0%, while the majority are engaged in alternative forms of employment, such as business owners, government officers, and others. The average blood pressure was 115.2/74.7 mmHg, and the hematocrit was 35.8%. OGTT levels decreased throughout the 1, 2, and 3 h averages, which were 158.5, 133.9, and 120.1 mg/dL, respectively, whereas FPG averaged 85.4 mg/dL. Most women had negative urine tests for protein and glucose.

### 3.2. Trace and Toxic Element Concentrations

The mean concentrations of trace and toxic elements are shown in Table 3. According to the trace element levels group, the largest concentration is found in Fe, followed by Zn and Cu, which have respective amounts of 75,178.00 ± 12,045.00 µg/dL, 1187.20 ± 211.38 µg/dL, and 294.72 ± 67.19 µg/dL. Among all the elements examined, As and Cd had the lowest concentrations, while Pb had the highest concentration, averaging 5.59 ± 1.61 µg/dL. Cr came in second with an average of 2.80 ± 1.47 µg/dL.

The relationships among Cu, Zn, Fe, As, Cd, Cr, and Pb in maternal blood are presented in Table 4. Synergistic associations were observed between Fe and Cu (r_s_ = 0.258), Zn and Cu (r_s_ = 0.286), as well as between Pb and Zn (r_s_ = 0.244), Fe (r_s_ = 0.198), As (r_s_ = 0.200), Cd (r_s_ = 0.200), and Cr (r_s_ = 0.168), respectively. In maternal blood, most correlations were weak (r < 0.399), except for Fe and Zn (r_s_ = 0.485), which demonstrated a moderate association. All primary toxic elements in blood showed statistically significant Spearman rank correlation values (*p* < 0.05).

### 3.3. Correlation Coefficients of Selected Toxic Elements and Biological Parameters

Table 5 displays the correlations between main toxic element levels and socio-demographic traits. We found modest but statistically significant correlations between biological indicators and toxic element levels, as indicated by the Spearman rank correlation values. The blood concentration of Cu was significantly correlated with hematocrit (r_s_ = 0.174, *p* < 0.05). The level of Zn was significantly correlated with the hematocrit (r_s_ = −0.188, *p* < 0.01), OGTT 2 h (r_s_ = −0.174, *p* < 0.05), and OGTT 3 h (r_s_ = −0.220, *p* < 0.01), respectively. As and Cd levels were correlated with urine glucose (r_s_ = −0.184, *p* < 0.01); the level of Cr was correlated with FPG (r_s_ = −0.141, *p* < 0.05); the level of Pb was significantly associated with the hematocrit (r_s_ = −0.219, *p* < 0.01); and the level of Fe was significantly associated with the hematocrit (r_s_ = 0.274, *p* < 0.01) and OGTT 2 h (r_s_ = 0.162, *p* < 0.05). All these relationships were statistically significant.

The median and min–max values of trace and toxic elements across trimesters are presented in Table 6. Kruskal–Wallis tests showed no significant differences for As, Cd, and Cr, whereas Pb, Cu, Fe, and Zn demonstrated significant variation across trimesters (*p* < 0.05).

The Kruskal–Wallis test revealed significant differences in Cu, Fe, Zn, and Pb concentrations across trimesters in Table 7. Post hoc pairwise comparisons using Dunn’s test with Bonferroni correction showed that for Cu, trimester 1 and trimester 3 (*p* < 0.001), as well as trimester 2 and trimester 3 (*p* = 0.038), differed significantly. For Fe, trimester 1 significantly differed from trimester 2 (*p* = 0.013) and trimester 3 (*p* = 0.005). For Zn, a significant difference was observed between trimester 2 and trimester 3 (*p* = 0.021). For Pb, trimester 1 differed significantly from trimester 2 (*p* = 0.040).

## 4. Discussion

In the present study, we measured blood levels of nutritionally essential trace elements, Cu, Zn, and Fe together the toxicants, Cd, Pb, Cr, and As in 200 pregnant Thai women, aged 18 to 45 years (mean age 30.2). They all resided in the southern region of Thailand. We then tested for the association of these blood elements with various indicators of maternal health.

The respective mean blood concentrations of Cu, Zn, and Fe in study subjects of 294.723, 1187.20, and 75,178.00 µg/dL were within the recommended reference ranges for pregnant women of 200–300, 700–1500, and 62,000–80,200 µg/dL for Cu, Zn and Fe, respectively [21]. The favorable status for essential metals, Cu, Zn, and Fe could be attributable to Thailand’s nationwide comprehensive prenatal care program, consisting of early antenatal registration (12 weeks prior to gestation), routine risk assessment, hematologic testing, universal iron and folic acid supplementation, recommendations for iodine and calcium intake, and regular follow-up visits to monitor maternal and fetal health [22].

Additional contributing factors were a high proportion (88%) of participants with at least a secondary education, and the young age group with an access to digital media. This demographic profile aligns with evidence that younger, more educated women are more likely to seek pregnancy-related health information through online platforms such as websites, blogs, apps, and social media, thereby enhancing their ability to access, interpret, and apply self-care guidance [23]. Our data are consistent with a study in China, which reported sufficient blood Zn levels in 92.2% of 3187 pregnant women [24]. In comparison, due to low dietary diversity, poverty, and limited health service utilization, the prevalence rates for Zn, Fe, and Cu deficiencies in rural Ethiopia were as high as 80.9%, 62.1% and 71.9%, respectively [25]. Despite the observed favorable status of essential metals, mean blood Pb concentration of 5.59 µg/dL was higher than the reference value (<5 µg/dL, ≈0.5 µg/L) [26]. Mean values for blood As (1.87 µg/L) and Cd (0.98 µg/L) were below international thresholds (As: <10 µg/L; Cd: <1 µg/L), while mean blood Cr (2.80 µg/L) was within the acceptable range [27,28,29].

Data on blood concentrations of nutritionally essential metals (Cu, Zn, and Fe) and toxicants (Pb, Cd, As, and Cr) study raised a concern about excessive Pb exposure in a significant proportion of participants in the present study. The source of Pb exposure among them should be an area for future research, given that one-third of pregnant women worldwide are at risk of Fe deficiency [30,31] and Pb exposure appeared to be widespread globally. Elevated blood Pb, Cd, and As concentrations in low- and middle-income countries, have often been linked to contaminated water, dietary sources, and mining activities [32,33,34]. In high-income countries, seafood consumption may contribute to high blood Pb levels [35,36].

Consistent with a previous study by Hegazy et al. [37], lower hematocrit levels correlated with higher blood Pb levels. This might be due to an effect of Pb on heme biosynthesis through inhibiting the enzyme δ-aminolevulinic acid dehydratase. As a result of reduced hemoglobin production, the hematocrit level is lowered, which may impair oxygen delivery to the fetus [38]. Comparable findings from studies in China and South Africa suggest that maternal exposure to Pb and Cd is associated with anemia, impaired fetal growth, and abnormal glucose metabolism [39,40]. Sufficient intake levels of Zn and Fe decreased risks of low birth weight and preeclampsia [41]. An inverse relationship between hematocrit and Pb may have a significant consequence in populations and may be of utility for screening anemia prevalence [42]. These findings highlight the importance of considering both toxic and essential elements in pregnancy research [43].

Herein, we have observed variations in blood elemental composition that were trimester specific. Blood Pb levels increased in the first trimester but declined in the second, while blood Cu levels showed a steady increase across pregnancy. Blood Fe concentrations fell after the first trimester, whereas blood Zn increased slightly in the third trimester. These patterns appeared to be consistent with the physiological changes during pregnancy, like expanded plasma volume, increased metabolic demands, and altered micronutrient utilization [44]. The similar trimester-dependent trends have been observed in Chinese and U.S. cohorts, where blood Cu increased progressively during pregnancy [45,46], Fe levels declined after the first trimester [47,48], with Pb showed dynamic changes across gestation [49,50]. Such temporal variations indicate that the gestational stage is crucial for interpreting biomarker levels and highlight the need for longitudinal monitoring across pregnancy.

The present study has addressed a key knowledge gap regarding multiple toxics and trace element levels among pregnant women in Thailand. It contributed region-specific evidence to the growing literature. These findings are relevant to Thailand and other regions facing similar challenges. Strengthening prenatal care programs with routine monitoring of toxicant exposure and nutritional status may help mitigate risks, improve maternal health, and support healthier pregnancy outcomes. However, the study has certain limitations, including the use of blood as the sole biomarker, which primarily reflects recent rather than cumulative exposure, and the absence of repeated measurements across trimesters that could capture temporal variations in trace and toxic element levels during pregnancy. In addition, the lack of detailed dietary and environmental data may limit the interpretation of exposure sources. Future studies with longitudinal follow-up, trimester-specific sampling, multiple biomarkers, and comprehensive exposure assessments are warranted to strengthen causal inference and clarify dynamic changes that occur during pregnancy. Additional study is required to obtain further details.

## 5. Conclusions

The present study provides data on blood concentrations of the essential metals Cu, Zn, and Fe in 200 pregnant women, aged 18 to 45 years, who resided in southern Thailand. It also provides blood concentrations of the toxic elements Cd, Pb, Cr, and As. Adverse effects of Pb on hematocrit levels have been observed together with protective effects of Cu and Fe. Dietary sources of Pb among participants require further research, as does the strategy to minimize the intestinal absorption of Pb.

## Figures and Tables

**Table 1:** The analytical method parameters of ICP-OES.

Elements	Spectral Line (nm)	Plasma Torch Position	%RSE	R2
Cu	327.395	Axial	8.78	0.99964
Zn	213.857	Axial	8.00	0.99964
Fe	238.204	Axial	8.45	0.99972
As	193.696	Axial	10.93	0.99993
Cd	214.439	Axial	1.62	0.99999
Pb	220.353	Axial	1.57	0.99995
Cr	267.716	Axial	1.23	0.99999

Cu: Cupper; Zn: Zinc; Fe: Iron; As: Arsenic; Cd: Cadmium; Pb: Lead; Cr: Chromium.

**Table 2:** Socio-demographic characteristics of pregnant women.

Variables	Mean ± SD or *n* (%)			
	Total (*n* = 200)	1st Trimester (*n* = 71)	2nd Trimester (*n* = 47)	3rd Trimester (*n* = 82)
Age (years)	30.2 ± 7.5	30.7 ± 1.0	30.2 ± 1.2	29.8 ± 0.7
G-BMI (kg/m^2^)	27.5 ± 5.9	27.66 ± 0.8	25.77 ± 0.8	28.32 ± 0.6
Education				
Less than high school	24 (12.0)	10 (14.1)	4 (8.9)	10 (11.9)
High school	104 (52.0)	37 (52.1)	27 (60.0)	40 (47.6)
Higher education	72 (36.0)	24 (33.8)	14 (31.1)	34 (40.5)
Gravidity				
1	60 (30.0)	21 (29.6)	12 (26.7)	27 (32.1)
2	51 (25.5)	20 (28.2)	10 (22.2)	21 (25.0)
≥3	89 (44.5)	30 (42.3)	23 (51.1)	36 (42.9)
Maternal occupation				
Housewives	66 (33.0)	25 (35.2)	18 (40.0)	23 (27.4)
Other	134 (67.0)	46 (64.8)	27 (60.0)	61 (72.6)
SBP (mmHg)	115.2 ± 12.2	116.7 ± 1.3	113.8 ± 1.9	114.7 ± 1.4
DBP (mmHg)	74.7 ± 9.9	77.0 ± 0.9	73.8 ± 1.3	73.3 ± 1.3
Hematocrit (%)	35.8 ± 3.6	36.3 ± 0.4	35.5 ± 0.5	35.6 ± 0.4
Plasma glucose				
FPG (mg/dL)	85.4 ± 18.5	90.1 ± 2.9	85.0 ± 2.8	87.7 ± 1.0
OGTT 1 h (mg/dL)	158.5 ± 67.1	175.1 ± 11.8	150.2 ± 6.9	148.8 ± 3.4
OGTT 2 h (mg/dL)	133.9 ± 39.9	140.0 ± 5.3	133.2 ± 7.3	129.2 ± 3.1
OGTT 3 h (mg/dL)	120.1 ± 33.8	117.9 ± 4.5	121.9 ± 6.7	120.8 ± 2.4
Urine Protein				
Negative	184 (92.0)	62 (89.9)	43 (95.6)	77 (91.7)
Positive	16 (8.0)	7 (10.1)	2 (4.4)	7 (8.3)
Urine Glucose				
Negative	186 (93.0)	64 (90.1)	42 (93.3)	80 (95.2)
Positive	14 (7.0)	7 (9.9)	3 (6.6)	4 (4.8)

GBMI: Gestational body mass index; SBP: Systolic blood pressure; DBP: Diastolic blood pressure; FPG: Fasting plasma glucose; OGTT: Oral glucose tolerance test; SD: standard deviation.

**Table 3:** Trace and toxic element concentrations (µg/dL).

Blood Elements	Mean	SD	Range
Trace elements			
Cu	294.72	67.19	145.00–525.00
Zn	1187.20	211.38	479.00–1971.00
Fe	75,178.00	12,045.00	41,713.00–101,887.00
Toxic elements			
As	1.87	1.43	ND–6.00
Cd	0.98	0.27	ND–4.00
Pb	5.59	1.61	2.00–11.00
Cr	2.80	1.47	1.00–16.00

SD: standard deviation; ND: not detected; Cu: Cupper; Zn: Zinc; Fe: Iron; As: Arsenic; Cd: Cadmium; Pb: Lead; Cr: Chromium.

**Table 4:** Correlations between blood toxic element concentrations (Spearman’s rho).

	Cu	Zn	Fe	As	Cd	Cr	Pb
Cu	1.000						
Zn	0.286 **	1.000					
Fe	0.258 **	0.485 **	1.000				
As	−0.159 *	−0.074	−0.151 *	1.000			
Cd	−0.159 *	−0.074	−0.151 *	1.000 **	1.000		
Cr	0.047	0.051	0.047	0.094	0.094	1.000	
Pb	0.110	0.244 **	0.198 **	0.200 **	0.200 **	0.168 *	1.000

** Correlation is significant at the 0.01 level (2-tailed), * Correlation is significant at the 0.05 level (2-tailed). Cu: Cupper; Zn: Zinc; Fe: Iron; As: Arsenic; Cd: Cadmium; Cr: Chromium; Pb: Lead.

**Table 5:** Correlation between blood element concentrations and health effects in pregnant women.

Variables	Correlation Coefficient						
	Cu	Zn	Fe	As	Cd	Cr	Pb
GBMI	0.023	0.028	0.051	0.049	0.049	0.051	0.121
SBP	−0.074	0.026	0.097	0.037	0.037	0.003	0.044
DBP	−0.099	0.022	0.122	0.067	0.067	−0.024	0.085
Hematocrit	0.174 *	−0.188 **	0.274 **	0.093	0.093	−0.031	−0.219 **
Urine protein	−0.079	−0.084	−0.003	0.032	0.032	−0.049	−0.006
Urine glucose	−0.126	−0.036	0.064	−0.184 **	−0.184 **	−0.126	−0.016
FPG	−0.162*	−0.030	0.054	0.043	0.043	−0.141 *	0.046
OGTT 1 h	−0.038	0.119	0.095	0.032	0.032	0.005	−0.060
OGTT 2 h	−0.026	−0.174 *	0.162 *	0.023	0.023	0.121	−0.016
OGTT 3 h	0.101	−0.220 **	0.110	0.006	0.006	0.072	−0.053

** Correlation is significant at the 0.01 level (2-tailed), * Correlation is significant at the 0.05 level (2-tailed). G-BMI: Gestational body mass index; SBP: Systolic blood pressure; DBP: Diastolic blood pressure; FPG: Fasting plasma glucose; OGTT: Oral glucose tolerance test.

**Table 6:** Blood concentrations of trace and toxic elements in pregnant women across trimesters [Median (Min–Max)].

Blood Elements (µg/dL)	1st Trimester (*n* = 71)	2nd Trimester (*n* = 47)	3rd Trimester (*n* = 82)	*p*-Value
Trace elements				
Cu	265.0 (145.0–442.0)	288.0 (149.0–402.0)	326.5 (154.0–525.0)	<0.001 **
Zn	1209.0 (479.0–1549.0)	1124.0 (715.0–1607.0)	1234.5 (708.0–1971.0)	0.026 *
Fe	81,780.0 (48,256.0–101,887.0)	75,204.0 (43,462.0–96,510.0)	73,022.0 (41,713.0–101,578.0)	0.002 *
Toxic elements				
As	2.0 (ND–6.0)	2.0 (ND–5.0)	1.0 (ND–5.0)	0.531
Cd	1.0 (ND–1.0)	1.0 (ND–1.0)	1.0 (ND–4.0)	0.739
Cr	3.0 (1.0–4.0)	3.0 (2.0–16.0)	3.0 (2.00–6.0)	0.691
Pb	6.0 (1.0–9.0)	5.0 (1.0–9.0)	6.0 (2.0–11.0)	0.033 *

**. Correlation is significant at the 0.01 level (2-tailed), *. Correlation is significant at the 0.05 level (2-tailed). Cu: Cupper; Zn: Zinc; Fe: Iron; As: Arsenic; Cd: Cadmium; Cr: Chromium; Pb: Lead.

**Table 7:** Post hoc pairwise comparisons of blood concentration of trace and toxic elements in pregnant women across trimesters.

Blood Elements	Pairwise Trimester Comparison	Test Statistic	Std. Error	Z	*p*-Value	Adj. *p*-Value
Cu	1 vs. 2	–19.741	10.883	–1.814	0.070	0.209
	1 vs. 3	–46.122	9.382	–4.916	<0.001	<0.001 *
	2 vs. 3	–26.382	10.589	–2.492	0.013	0.038 *
Fe	2 vs. 3	–1.369	10.589	–0.129	0.897	1.000
	2 vs. 1	30.993	10.884	2.848	0.004	0.013 *
	3 vs. 1	29.625	9.383	3.157	0.002	0.005 *
Zn	2 vs. 1	17.983	10.884	1.652	0.098	0.295
	2 vs. 3	–28.617	10.589	–2.703	0.007	0.021 *
	1 vs. 3	–10.634	9.383	–1.133	0.257	0.771
Pb	2 vs. 3	–22.631	10.388	–2.179	0.029	0.088
	2 vs. 1	26.395	10.677	2.472	0.013	0.040 *
	3 vs. 1	3.765	9.204	0.409	0.683	1.000

* Correlation is significant at the 0.05 level (2-tailed); Cu: Cupper; Fe: Iron, Zn: Zinc’ Pb: Lead.

## Data Availability

The data supporting the findings of this study are available from the corresponding author upon reasonable request.

## Abbreviations

Cu	Copper
Zn	Zinc
Ca	Calcium
Mg	Magnesium
Fe	Iron
Pb	Lead
Cd	Cadmium
Cr	Chromium
As	Arsenic
Hg	Mercury
G-BMI	Gestational body mass index
CBC	Complete blood count
FPG	fasting plasma glucose
OGTT	Oral glucose tolerance testing
SBP	Systolic blood pressure
DBP	Diastolic blood pressure
G	Gravida
ICP-OES	Inductively coupled plasma optical emission spectroscopy
LoD	Limit of detection
